# Medical residents’ knowledge, attitudes and practices regarding antibiotics, antimicrobial stewardship and multidrug-resistant bacteria: a cross-sectional study in a major university in Iran

**DOI:** 10.3389/fmed.2024.1435542

**Published:** 2024-09-23

**Authors:** Fatemeh Kiani, Ghazaleh Sajadi, Narges Motamedi, Mehrzad Salmasi, Hamid Solgi

**Affiliations:** ^1^Resident of Internal Department, Isfahan University of Medical Sciences, Isfahan, Iran; ^2^Department of Internal Medicine, School of Medicine, Isfahan University of Medical Sciences, Isfahan, Iran; ^3^Department of Community Medicine, School of Medicine, Isfahan University of Medical Sciences, Isfahan, Iran; ^4^Isfahan Endocrine and Metabolism Research Center, Isfahan University of Medical Sciences, Isfahan, Iran; ^5^Amin Hospital, Isfahan University of Medical Sciences, Isfahan, Iran

**Keywords:** knowledge-attitude-practice, medical residents, antimicrobial stewardship (AMS), multidrug-resistant (MDR) bacteria, Iran

## Abstract

**Background:**

Antimicrobial resistance (AMR) is one of the biggest threats to global public health systems. This study aimed to assess the knowledge, attitudes and practice about AMR, antimicrobial stewardship programs (ASPs) and multidrug-resistant (MDR) bacteria.

**Methods:**

A web-based questionnaire survey was conducted among the residents of Isfahan University of Medical Sciences from May to November 2023. Data analysis was done using SPSS version 24.0 software.

**Results:**

Overall, 400 out of 450 medical residents responded to the questionnaire, giving a response rate of 88.9%. The participants’ ages ranged from 26 to 54 years, and the majority were female (227/400 56.8%). Average scores for knowledge, attitudes, and practices were 53.70 ± 15.88, 36.97 ± 5.89 and 24.69 ± 4.24, respectively. In terms of knowledge, only 26.8% had heard the term “ASPs” and knew what it was. Most incorrect answers appeared to the treatment of infection caused by MDR bacteria including ESBL-producing *Escherichia coli* (27.8%) and carbapenem-resistant *Klebsiella pneumoniae* (30.8%), as well as the atypical bacteria (45.5%). Approximately, 50 and 71.7% said they had received no specific training in the fields of microbiological sampling methods and the appropriate time to prescribe antibiotics, respectively. Surprisingly, regarding practice, 81.8% of the respondents stated that antibiotics are used to treat flu or the common cold.

**Conclusion:**

Residents considered their training on important issues including ASPs, MDR bacteria and the spectrum of antibiotics insufficient. This result highlights the need for targeted training interventions about antibiotic prescription in the curriculum at the university with more emphasis on ASPs to limit the development of resistance.

## Introduction

Antimicrobial resistance (AMR) is one of the most serious public health concerns worldwide, posing a threat to the effective treatment of infectious diseases. AMR results in longer hospital stays, higher healthcare costs, higher mortality and higher medical resource consumption especially in low-income countries (1). It is estimated that a failure to address resistance to antibiotics would lead to approximately 10 million unnecessary deaths every year by 2050, resulting in projected economic costs as high as USD 100 trillion worldwide (2). The main factors associated with AMR in third-world countries include lack of sufficient knowledge and experience of clinicians, indeterminate diagnosis, inadequate dosage, use of antibiotics for non-bacterial infections, self-medication, over-prescription, incomplete treatment courses as well as poor infection control measures to prevent the spread of multidrug-resistant (MDR) bacteria in the hospitals (3, 4).

Iran has one of the highest rates of antibiotic consumption and resistance worldwide (5). The possible causes for this high consumption are related to the lack of knowledge about AMR, MDR pathogens and antimicrobial stewardship programs (ASPs), as well as the attitude and practices of physicians. Several knowledge, attitudes, and practices (KAP) surveys on antimicrobial resistance have been conducted among physicians, nurses and university students in both tertiary care and community settings (6–9). Understanding the KAP of the medical residents regarding antibiotics, antimicrobial stewardship and AMR is essential to developing targeted interventions and promoting appropriate antibiotic prescribing. Medical residents play a key role in the area of AMR when considering the inappropriate use or misuse of these agents. This is significantly more important when they are in charge of the administration and prescription of antibiotics in the hospitals and clinics where they work. Therefore, this population plays an important role in designing strategies that allow increasing awareness about the correct and rational use of antibiotics through communication and education.

In Iran, studies have shown high levels of antibiotic resistance in hospitals (10, 11), but published data assessing the KAP of medical residents toward AMR are absent. The present study aimed to investigate the medical residents’ KAP regarding antibiotics, antibiotic use and AMR in Iran to identify knowledge gaps and tailored educational interventions that could lead to wise use of antimicrobials and reduce the emergence of MDR organisms and their consequences.

## Materials and methods

### Study design and settings

A cross-sectional analytic online-based study was conducted to assess the KAP of medical residents toward AMR and MDR organisms and ASPs in academic hospitals (Isfahan University of Medical Sciences) in the center of Iran between May to November 2023. During this period, the medical residents are rotated through the different hospitals, which are relevant for their respective medical specializations. A web-based survey software (Google Forms) was used to collect data and that was voluntary and anonymous.

### Study population

The Medical University of Isfahan (MUI) is one of the major public universities in Iran; every year, many specialists from all fields graduate from it. During the study period, the university had 450 medical residents. The sample size was determined by using Epi-info software. For a confidence level of 95%, the sample size equal to 450. The term “medical residents” referred to physicians after qualification from medical school who were still in their training years for a specialty. An electronic invitation letter including a link to the survey was sent to 450 residents from the first year to the fourth year in various specialties including Internal Medicine, Infectious Diseases, Orthopedic Surgery, General Surgery, Gynecology, Neurology, Neurosurgery, Urology, Cardiology, Pediatric, and ENT. During the period the survey remained open, medical residents received 3 reminders (email or call) to get the maximum number of participants. Due to the large number of subspecialties of residents and the meaningless analysis of each specialist separately, we had to categorize them into similar groups based on the amount of exposure of each resident’s group with antibiotic prescription. Our justification for such a division was our expectation of the similarity of antibiotic knowledge of these groups as follows:

Group 1 (Internalist, Infectious, Pediatric, *N* = 116): This group had the most exposure to the prescription of antibiotics. They were the group who visited many patients with medical problems every day. Group 2 (Cardiologist, Neurologist, *N* = 98): This group also faced medical problems in their patients, but they usually did not face the routine prescription of antibiotics. Group 3 (Orthopedist, Neurosurgeon, Urologist, ENT, *N* = 105): This group was the category of minor surgeries, which were usually faced with a limited field, and their patients experienced fewer complications from infection due to the limited surgical field. Group 4 (Gynecologist, Surgeon, *N* = 81): They were the group of major surgeries with extensive surgical sites.

### Exclusion criteria

Due to the lack of antibiotic prescription during the residency, groups such as radiology, psychology, physical medicine and rehabilitation, skin, nuclear, emergency medicine, anesthesia, ophthalmology, radio-oncology, and pathology were excluded from the study.

### Questionnaire design

A structured questionnaire was developed after a literature review of similar studies (12–14), and in consultation with relevant experts from the field of bacteriology and statistics. The questionnaire included two parts. In the first part, questions are about demographics and general information about residents, such as their Gender, year of study, and specialty. The second part had 31 questions including three sections: (1) 11 questions in a 2-level Likert scale to evaluate the knowledge of students about issues. In more detail, knowledge questions are related to identifying antibiotics, antibiotic role, antibiotic side effects, and awareness of antibiotic resistance. (2) The second section comprised 12 questions to assess the attitudes and perceptions toward AMR and MDR organisms and ASPs. (3) The last part of the questionnaire included eight questions on practices ranging from the participant’s awareness of the pattern of AMR at their workplace hospitals, proper use of international and local antibiotics guidelines, and consultation with infectious disease specialists. The attitude and practice questions have been designed on a 5-level Likert scale from 1 (completely disagree) to 5 (completely agree). The goal of the questionnaire was introduced to 450 voluntary participants and their agreement was reached before filling in the questionnaire. To evaluate the face validity of the questionnaire, five specialists assessed and commented on the appropriateness and clarity of the questions. The Content Validity Ratio (CVR) and the Content Validity Index (CVI) were used for the content validity analysis. The minimum acceptable value for CVR is 0.99 when the number of experts is five and the acceptable value for CVI is 0.79. The result of CVR and CVI shows all items are appropriate. The reliability of the questionnaire was assessed by calculating Cronbach’s alpha. The Cronbach’s alpha (0.711) demonstrated good reliability.

### Data analysis

Each survey response was considered valid if more than 80% of questions (25 or more) had been answered. After collecting valid responses, Data were analyzed by using SPSS version 24 software. Statistical analysis, including the descriptive method, Chi-square and Fisher’s exact test was implemented on the total answers of respondents. The relation of participants’ knowledge, attitude, and practice with their sociodemographic features (age, gender, specialty) were compared and analyzed using the Mann–Whitney U test and Kruskall-Wallis H test.

## Results

### Demographic characteristics

During the study period, out of 450 potential respondents, 400 responded and completed the questionnaires (response rate; 88.9%). Table 1 shows details on demographic characteristics of the study participants. Their age ranged from 26 to 54 years and 227 (56.8%) participants were females. The mean age of respondents was 30.90 ± 3.45 years and most of them, 229 (57.2%) were smaller or equal to 30 years. Participants were divided into four groups based on their field and the year of their university entrance, respectively. The largest group of respondents was group-1116 (29.0%), which included internal medicine, infectious, and pediatric residents. One hundred and four (26%) of the residents were in their first year of training, 105 (26.3%) in their second, 98 (24.6%) in their third, and 93 (23.3%) in their fourth or longer.

**Table 1:** Demographic characteristics of participants (*n* = 400).

Variables	Number (%)
Age (years)	
Mean = 30.90 ± 3.45	
≤30	229 (57.2)
>30	171 (42.8)
Gender	
Male	173 (43.2)
Female	227 (56.8)
Professional status	
Group 1 (Internalist, Infectious, Pediatric)	116 (29.0)
Group 2 (Cardiologist, Neurologist)	98 (24.5)
Group 3 (Orthopedist, Neurosurgeon, Urologist, ENT)	105 (26.3)
Group 4 (Gynecologist, Surgeon)	81 (20.2)
Years of training	
First Year	104 (26)
Second Year	105 (26.3)
Third Year	98 (24.6)
Fourth Year	93 (23.3)

### Medical residents’ knowledge

Data from 11 knowledge questions were analyzed according to two topics: (1) based on the professional status of participants, and (2) based on the participants’ years of entering the university. The responses to knowledge items were presented in Tables 1, 2. Overall, the average knowledge score among participants was 53.70 ± 15.88 in a 100-point scale. Approximately three-quarters of the participants (293/400, 73.2%) lacked awareness about the antimicrobial stewardship program in Iran.

**Table 2:** Knowledge based on the university entry year of 400 participants.

	Number (percentage) of respondents giving a correct answer					*p-values
	Total (*N* = 400)	First-year (*N* = 104)	Second-year (*N* = 105)	Third-year (*N* = 98)	Fourth-year (*N* = 93)	
Q1. Do you have any information about the ASP in Iran?	107 (26.8%)	25 (24%)	27 (25.7%)	29 (29.6%)	26 (28.0%)	0.764
Q2. Can clindamycin cover the MRSA?	222 (55.5%)	66 (64.1%)	51 (48.6%)	56 (57.1%)	49 (52.7%)	0.138
Q3. Can colistin cover gram-positive bacteria?	266 (66.5%)	68 (65.4%)	67 (63.8%)	65 (66.3%)	66 (71.0%)	0.736
Q4. Can fluoroquinolones cover the atypical bacteria?	182 (45.5%)	51 (49%)	49 (46.7%)	43 (43.9%)	39 (41.9%)	0.796
Q5. Can ceftazidime cover the *Pseudomonas aeruginosa*?	286 (71.5%)	72 (69.2%)	74 (70.5%)	70 (71.4%)	70 (75.3%)	0.793
Q6. Can the cefoxitin disc diffusion test detect MRSA?	205 (51.3%)	45 (43.3%)	57 (54.3%)	49 (50.0%)	54 (58.1%)	0.209
Q7. Poor infection control practices by healthcare professionals cause the spread of antimicrobial resistance.	312 (78%)	81 (77.9%)	83 (79.0%)	77 (78.6%)	71 (76.3%)	0.971
Q8. Can *Staphylococcus epidermidis* be reported as contamination in blood culture?	283 (70.8%)	79 (76%)	69 (65.7%)	67 (68.4%)	68 (73.1%)	0.386
Q9. In the patient’s blood culture who developed a fever after surgery and is receiving ceftriaxone, ESBL-producing *E-coli* bacteria grew. Will you change his/her antibiotic to meropenem?	111 (27.8%)	25 (24%)	33 (31.4%)	26 (26.5%)	27 (29.0%)	0.599
Q10. If carbapenem-resistant *K. pneumoniae* (MIC of meropenem ≥32 mg/L) is isolated from the BAL specimen, I will continue meropenem.	123 (30.8%)	33 (31.7%)	28 (26.7%)	35 (35.7%)	27 (29.0%)	0.540
Q11. I will prescribe colistin if a carbapenem-resistant *Proteus mirabilis* isolate has grown in the 75-year-old patient’s urine culture.	266 (66.5%)	66 (63.5%)	68 (64.8%)	63 (64.3%)	69 (74.2%)	0.340
Overall score (mean_SD)	53.70 ± 15.88	53.22 ± 16.51	52.46 ± 14.71	53.80 ± 15.42	55.32 ± 16.93	0.637

ASM, Antimicrobial stewardship program; MRSA, Methicillin-resistant *Staphylococcus aureus*; MDR, Multidrug-resistant; ESBL, Extended-spectrum beta-lactamases; MIC, Minimum inhibitory concentrations; VAP, Ventilator-associated pneumonia. *p*-values derived from Fisher’s exact tests or one-way analysis of variance.

The majority (286/400, 71.5%) of respondents correctly answered to appropriate coverage of ceftazidime to *Pseudomonas aeruginosa*. Nearly all the participants (312/400, 78%) expressed to importance of the infection control process in hospitals to decrease the spread of MDR bacteria. Most incorrect answers appeared to the treatment of blood infection caused by ESBL-producing *Escherichia coli* (Q9, 27.8%), followed by continuing the treatment of a patient with a lung infection caused by carbapenem-resistant *Klebsiella pneumoniae* with a MIC of meropenem ≥32 mg/L (Q10, 30.8%), and fluoroquinolone prescriptions for cover the atypical bacteria (Q4, 45.5%).

As it is described in Table 3, according to the *p*-values of Q4, 6 and 8, there is a remarkable difference between answers of four groups. Regarding the question of covering atypical bacteria with fluoroquinolones, the highest and lowest percentage of correct answers belonged to groups 1 (68/116, 58.6%) and 3 (30/105, 28.6%), respectively. Also, regarding the use of cefoxitin disc diffusion test to detect methicillin-resistant *Staphylococcus aureus* (MRSA), the lowest (35.3%) and highest (62.9%) correct answers were observed in groups 3 and 1, respectively. A notable percentage of group 1 (87.9%) selected the correct answer to the question about the possibility of blood culture contamination with *Staphylococcus epidermidis*, and the lowest percentage of correct answers to this question belonged to group 4 with 55%. More than half of the participants were mindful of the coverage of colistin and its intrinsic resistance to some bacterial strains like *Proteus mirabilis.*

**Table 3:** Knowledge based on professional status of 400 participants.

	Number (percentage) of respondents giving a correct answer					*p-values
	Total (*N* = 400)	Group 1 (*N* = 116)	Group 2 (*N* = 98)	Group 3 (*N* = 105)	Group 4 (*N* = 81)	
Q1. Do you have any information about the ASP in Iran?	107 (26.8%)	31 (26.7%)	20 (20.4%)	30 (28.6%)	26 (32.1%)	0.338
Q2. Can clindamycin cover the MRSA?	222 (55.5%)	55 (47.4%)	53 (54.1%)	66 (62.9%)	48 (59.3%)	0.116
Q3. Can colistin cover gram-positive bacteria?	266 (66.5%)	69 (59.5%)	62 (63.3%)	80 (76.2%)	55 (67.9%)	0.057
Q4. Can fluoroquinolones cover the atypical bacteria?	182 (45.5%)	68 (58.6%)	50 (51.0%)	30 (28.6%)	34 (42.0%)	**<0.001**
Q5. Can ceftazidime cover the *Pseudomonas aeruginosa*?	286 (71.5%)	90 (77.6%)	71 (72.4%)	68 (64.8%)	57 (70.4%)	0.209
Q6. Can the cefoxitin disc diffusion test detect MRSA?	205 (51.3%)	41 (35.3%)	51 (52.0%)	66 (62.9%)	47 (58.0%)	**<0.001**
Q7. Poor infection control practices by healthcare professionals cause the spread of antimicrobial resistance.	312 (78.0%)	93 (80.2%)	74 (75.5%)	81 (77.1%)	64 (79.0%)	0.857
Q8. Can *Staphylococcus epidermidis* be reported as contamination in blood culture?	283 (70.8%)	102 (87.9%)	70 (71.4%)	66 (62.9%)	45 (55.6%)	**<0.001**
Q9. In the patient’s blood culture who developed a fever after surgery and is receiving ceftriaxone, ESBL-producing *E-coli* bacteria grew. Will you change his/her antibiotic to meropenem?	111 (27.8%)	39 (33.6%)	25 (25.5%)	25 (23.8%)	22 (27.2%)	0.381
Q10. If carbapenem-resistant *K. pneumoniae* (MIC of meropenem ≥32 mg/L) is isolated from the BAL specimen, I will continue meropenem.	123 (30.8%)	42 (36.2%)	30 (30.6%)	26 (24.8%)	25 (30.9%)	0.335
Q11. I will prescribe colistin if a carbapenem-resistant *Proteus mirabilis* isolate has grown in the 75-year-old patient’s urine culture.	266 (66.5%)	76 (65.5%)	60 (61.2%)	70 (66.7%)	60 (74.1%)	0.339
Overall score (mean _ SD)	53.70 ± 15.88	55.32 ± 16.10	52.50 ± 15.08	52.64 ± 16.51	54.20 ± 15.70	0.509

MRSA, Methicillin-resistant *Staphylococcus aureus*; ESBL, Extended-spectrum beta-lactamases; MIC, Minimum inhibitory concentrations; VAP, Ventilator-associated pneumonia. *p*-values derived from Fisher’s exact tests or one-way analysis of variance.Bold values represent the statistically significant results.

### Medical residents’ attitude

Data from 12 attitude questions were analyzed according to two topics: (1) based on the professional status of participants, and (2) based on the participants’ years of entering the university.

Tables 4, 5 present the levels of attitudes toward AMR and MDR organisms and ASPs among the study participants. The average attitude score among participants was 36.97 ± 5.89 on a 60-point scale.

**Table 4:** Attitudes based on the university entry year of 400 participants.

	First-year (*N* = 104)			Second-year (*N* = 105)			Third-year (*N* = 98)			Fourth-year (*N* = 93)			*p*-values
	A [N (%)]	B [N (%)]	C [N (%)]	A [N (%)]	B [N (%)]	C [N (%)]	A [N (%)]	B [N (%)]	C [N (%)]	A [N (%)]	B [N (%)]	C [N (%)]	
Q1. Can duration change of antibiotic prescription cause antibiotic resistance?	86	6	12	90	3	12	82	2	14	81	3	9	0.763
	82.7%	5.8%	11.7%	85.7%	2.9%	11.4%	83.7%	2.0%	14.3%	87.1%	3.2%	9.7%	
Q2. A 30-year-old woman is presented with a complaint of dysuria and frequency. Do you hospitalize her and prescribe ceftriaxone?	82	4	18	81	10	14	77	9	12	66	12	15	0.381
	78.8%	3.9%	17.5%	77.1%	9.5%	13.3%	78.6%	9.2%	12.2%	71.0%	12.9%	16.1%	
Q3. A 42-year-old man was presented with complaints of sore throat, headache, and runny nose. Do you prescribe azithromycin?	88	5	11	80	8	17	72	14	12	72	12	9	0.178
	84.7%	4.9%	10.7%	76.2%	7.6%	16.2%	73.5%	14.3%	12.2%	77.4%	12.9%	9.7%	
Q4. Is antibiotic usage excessive and inappropriate in Iran?	88	7	9	87	5	13	76	7	15	69	8	16	0.546
	84.7%	6.8%	8.7%	82.9%	4.8%	12.4%	77.6%	7.1%	15.3%	74.2%	8.6%	17.2%	
Q5. Is antibiotic usage excessive and inappropriate in your hospital?	87	2	15	95	3	7	82	7	9	79	6	8	0.208
	83.7%	1.9%	14.6%	90.5%	2.9%	6.7%	83.7%	7.1%	9.2%	84.9%	6.5%	8.6%	
Q6. Is the health of you and your family affected by antibiotic resistance?	88	6	10	79	8	18	83	4	11	73	5	15	0.553
	84.7%	5.8%	9.7%	75.2%	7.6%	17.1%	84.7%	4.1%	11.2%	78.5%	5.4%	16.1%	
Q7. Can inappropriate and misuse of antimicrobials cause antimicrobial resistance?	92	3	9	89	5	11	85	4	9	78	2	13	0.830
	88.5%	2.9%	8.7%	84.8%	4.8%	10.5%	86.7%	4.1%	9.2%	83.9%	2.2%	14.0%	
Q8. Have you been educated enough on how to take microbiological samples during your study at the university?	59	9	36	54	14	37	53	15	30	34	9	50	0.022
	56.5%	8.7%	35.0%	51.4%	13.3%	35.2%	54.1%	15.3%	30.6%	36.6%	9.7%	53.8%	
Q9. Have you been educated enough on how to interpret the antibiograms during your study at the university?	64	18	22	74	10	21	55	15	28	47	17	29	0.113
	61.4%	17.5%	21.4%	70.5%	9.5%	20.0%	56.1%	15.3%	28.6%	50.5%	18.3%	31.2%	
Q10. Have you been educated enough about the appropriate time for initiation of antibiotics during your study at the university?	24	13	67	31	12	62	32	12	54	26	14	53	0.741
	22.5%	12.6%	65.0%	29.5%	11.4%	59.0%	32.7%	12.2%	55.1%	28.0%	15.1%	57.0%	
Q11. Have you been educated enough about the appropriate time to switch the intravenous antibiotics to the oral equivalent during your study at the university?	64	11	29	61	11	33	56	12	30	51	16	26	0.818
	61.4%	10.7%	28.2%	58.1%	10.5%	31.4%	57.1%	12.2%	30.6%	54.8%	17.2%	28.0%	
Q12. Have you been educated enough about the mechanisms of antibiotic resistance during your study at the university?	60	16	28	64	9	32	63	10	25	54	16	23	0.515
	57.5%	15.5%	27.2%	61.0%	8.6%	30.5%	64.3%	10.2%	25.5%	58.1%	17.2%	24.7%	
Overall score (mean _ SD)	36.79 ± 5.70			36.74 ± 6.23			37.01 ± 6.00				37.38 ± 5.64		0.873

A, Strongly agree or Agree; B, Neither agree nor disagree; C, Disagree or Strongly disagree.

**Table 5:** Attitudes based on professional status of 400 participants.

	Group 1 (*N* = 116)			Group 2 (*N* = 98)			Group 3 (*N* = 105)			Group 4 (*N* = 81)			*p*-values
	A [N (%)]	B [N (%)]	C [N (%)]	A [N (%)]	B [N (%)]	C [N (%)]	A [N (%)]	B [N (%)]	C [N (%)]	A [N (%)]	B [N (%)]	C [N (%)]	
Q1. Can duration change of antibiotic prescription cause antibiotic resistance?	101	3	12	79	3	16	88	5	12	71	3	7	0.704
	87.1%	2.6%	10.3%	80.6%	3.1%	16.3%	83.8%	4.8%	11.4%	87.7%	3.7%	8.6%	
Q2. A 30-year-old woman is presented with a complaint of dysuria and frequency. Do you hospitalize her and prescribe ceftriaxone?	99	6	11	78	9	11	76	12	17	53	8	20	**0.028**
	85.3%	5.2%	9.5%	79.6%	9.2%	11.2%	72.4%	11.4%	16.2%	65.4%	9.9%	24.7%	
Q3. A 42-year-old man was presented with complaints of sore throat, headache, and runny nose. Do you prescribe azithromycin?	103	6	7	75	7	16	78	15	12	56	11	14	**0.031**
	88.8%	5.2%	6.0%	76.5%	7.1%	16.3%	74.3%	14.3%	11.4%	69.1%	13.6%	17.3%	
Q4. Is antibiotic usage excessive and inappropriate in Iran?	101	4	11	69	9	20	79	8	18	71	6	4	**0.031**
	87.1%	3.4%	9.5%	70.4%	9.2%	20.4%	75.2%	7.6%	17.1%	87.7%	7.4%	4.9%	
Q5. Is antibiotic usage excessive and inappropriate in your hospital?	100	5	11	82	5	11	90	3	12	71	5	5	0.821
	86.2%	4.3%	9.5%	83.7%	5.1%	11.2%	85.7%	2.9%	11.4%	87.7%	6.2%	6.2%	
Q6. Is the health of you and your family affected by antibiotic resistance?	92	7	17	76	7	15	87	4	14	68	5	8	0.867
	79.3%	6.0%	14.7%	77.6%	7.1%	15.3%	82.9%	3.8%	13.3%	84.0%	6.2%	9.9%	
Q7. Can inappropriate and misuse of antimicrobials cause antimicrobial resistance?	102	3	11	82	4	12	88	5	12	72	2	7	0.911
	87.9%	2.6%	9.5%	83.7%	4.1%	12.2%	83.8%	4.8%	11.4%	88.9%	2.5%	8.6%	
Q8. Have you been educated enough on how to take microbiological samples during your study at the university?	72	11	33	52	12	34	43	11	51	33	13	35	**0.018**
	62.1%	9.5%	28.4%	53.1%	12.2%	34.7%	41.0%	10.5%	48.6%	40.7%	16.0%	43.2%	
Q9. Have you been educated enough on how to interpret the antibiograms during your study at the university?	72	14	30	65	12	21	57	17	31	46	17	18	0.392
	62.1%	12.1%	25.9%	66.3%	12.2%	21.4%	54.3%	16.2%	29.5%	56.8%	21.0%	22.2%	
Q10. Have you been educated enough about the appropriate time for initiation of antibiotics during your study at the university?	38	12	66	21	9	68	29	18	58	24	12	45	0.225
	32.8%	10.3%	56.9%	21.4%	9.2%	69.4%	27.6%	17.1%	55.2%	29.6%	14.8%	55.6%	
Q11. Have you been educated enough about the appropriate time to switch the intravenous antibiotics to the oral equivalent during your study at the university?	58	19	39	70	11	17	61	13	31	43	7	31	**0.022**
	50.0%	16.4%	33.6%	71.4%	11.2%	17.3%	58.1%	12.4%	29.5%	53.1%	8.6%	38.3%	
Q12. Have you been educated enough about the mechanisms of antibiotic resistance during your study at the university?	69	14	33	66	9	23	59	15	31	47	13	21	0.688
	59.5%	12.1%	28.4%	67.3%	9.2%	23.5%	56.2%	14.3%	29.5%	58.0%	16.0%	25.9%	

A, Strongly agree or Agree; B, Neither agree nor disagree; C, Disagree or Strongly disagree.Bold values represent the statistically significant results.

Nearly 86% (344/400) of the respondents agreed or strongly agreed that an inappropriate use of antimicrobials could cause antimicrobial resistance. The great majority of respondents (85.8%, 343/400) admitted that antibiotic prescriptions are excessive and inappropriate in their workplace hospitals. When participants were asked about getting enough information about the appropriate time to start antibiotics at the university, only 28.3% (113/400) of respondents agreed or strongly agreed that they were given enough information. When respondents were asked about how to take microbiological samples, exactly 50% (200/400) stated that they had not learned about microbiological sampling during their degree courses. As it is shown in Table 4, only Q8 has a *p*-value<0.05 and so in this question, there is a remarkable difference between answers of different years. As can be depicted from Table 5, according to the *p*-values (<0.05) of Q2, 3, 4, 8 and 11, there is a remarkable difference between the answers of the four groups.

### Medical residents’ practice

Data from eight practice questions were analyzed. Overall, the average practice score among participants was 24.69 ± 4.24 on a 40-point scale. Regarding practice, 81.8% (327/400) of the respondents stated that antibiotics are used to treat flu or the common cold, 78.3% (313/400) stated that antibiotics should be discontinued when symptoms disappear, 58.8% stated that they prescribe antibiotics because of the patient’s insistence, 62.3% (249/400) stated that they prescribe antibiotics according to the guidelines, 71.3% (285/400) stated that they prescribe antibiotics before preparing the microbiology culture results based on the local antibiotic resistance pattern, 65% (260/400) stated that before changing antibiotics, they differentiate colonization from infection based on the results of microbial culture, 80.3% (321/400) stated that before the antibiotic change, based on antibiogram result, they pay attention to the types of antibiotics the patient received previously and 70.5% they consult with infectious disease specialists to using of broad-spectrum antibiotics.

Table 6 shows only question 8 has a *p*-value <0.05 and so in this question, there is a significant difference between answers of different years of training. As shown in Table 7, questions 1, 2, 4, and 8 have *p*-value<0.05 and so in these questions there are significant differences between answers of different groups.

**Table 6:** Practices based on the university entry year of 400 participants.

	First-year (*N* = 104)			Second-year (*N* = 105)			Third-year (*N* = 98)			Fourth-year (*N* = 93)			*p*-values
	A [N (%)]	B [N (%)]	C [N (%)]	A [N (%)]	B [N (%)]	C [N (%)]	A [N (%)]	B [N (%)]	C [N (%)]	A [N (%)]	B [N (%)]	C [N (%)]	
Q1. Antibiotics are used to treat flu or the common cold.	88	5	11	88	8	9	79	8	11	72	6	15	0.688
	84.7%	4.9%	10.7%	83.8%	7.6%	8.6%	80.6%	8.2%	11.2%	77.4%	6.5%	16.1%	
Q2. Antibiotics should be discontinued when symptoms disappear	86	5	13	85	8	12	77	9	12	65	5	23	0.102
	82.7%	4.9%	12.6%	81.0%	7.6%	11.4%	78.6%	9.2%	12.2%	69.9%	5.4%	24.7%	
Q3. Do you prescribe antibiotics due to the patient’s insistence and to avoid discussion, despite the fact that the antibiotic is not necessary medically?	61	11	32	66	8	31	54	10	34	54	11	28	0.920
	58.5%	10.7%	31.1%	62.9%	7.6%	29.5%	55.1%	10.2%	34.7%	58.1%	11.8%	30.1%	
Q4. Do you prescribe antibiotics based on the guidelines recommended in your country and your own hospital?	65	13	26	64	8	33	67	7	24	53	8	32	0.482
	62.3%	12.6%	25.2%	61.0%	7.6%	31.4%	68.4%	7.1%	24.5%	57.0%	8.6%	34.4%	
Q5. Before receiving the culture results, do you prescribe empirical antibiotic therapy based on the common types of bacteria and the antibiotic resistance pattern of your hospital?	72	13	19	73	9	23	75	6	17	65	6	22	0.552
	69.2%	12.6%	18.4%	69.5%	8.6%	21.9%	76.5%	6.1%	17.3%	69.9%	6.5%	23.7%	
Q6. Can you distinguish colonization from infection based on antibiogram results?	66	14	24	69	18	18	59	8	31	66	7	20	0.870
	63.5%	13.6%	23.3%	65.7%	17.1%	17.1%	60.2%	8.2%	31.6%	71.0%	7.5%	21.5%	
Q7. To change the antibiotic regime due to the antibiogram, do you notice the history of receiving previous antibiotics?	84	10	10	82	9	14	80	5	13	75	6	12	0.862
	80.8%	9.7%	9.7%	78.1%	8.6%	13.3%	81.6%	5.1%	13.3%	80.6%	6.5%	12.9%	
Q8. To use broad-spectrum antibiotics, do you consult infectious disease specialists?	75	9	20	72	11	22	59	8	31	76	3	14	**0.037**
	72.1%	8.7%	19.4%	68.6%	10.5%	21.0%	60.2%	8.2%	31.6%	81.7%	3.2%	15.1%	
Overall score (mean _ SD)	24.57 ± 4.19			24.44 ± 4.30			24.51 ± 4.48			25.31 ± 3.96			0.454

A, Strongly agree or Agree; B, Neither agree nor disagree; C, Disagree or Strongly disagree.Bold values represent the statistically significant results.

**Table 7:** Practices based on professional status of 400 participants.

	Group 1 (*N* = 116)			Group 2 (*N* = 98)			Group 3 (*N* = 105)			Group 4 (*N* = 81)			*p*-values
	A [N (%)]	B [N (%)]	C [N (%)]	A [N (%)]	B [N (%)]	C [N (%)]	A [N (%)]	B [N (%)]	C [N (%)]	A [N (%)]	B [N (%)]	C [N (%)]	
Q1. Antibiotics are used to treat flu or the common cold.	107	2	7	80	5	13	78	12	15	62	8	11	**0.013**
	92.2%	1.7%	6.0%	81.6%	5.1%	13.3%	74.3%	11.4%	14.3%	76.5%	9.9%	13.6%	
Q2. Antibiotics should be discontinued when symptoms disappear	104	2	10	74	6	18	79	10	16	56	9	16	**0.014**
	89.7%	1.7%	8.6%	75.5%	6.1%	18.4%	75.2%	9.5%	15.2%	69.1%	11.1%	19.8%	
Q3. Do you prescribe antibiotics due to the patient’s insistence and to avoid discussion, despite the fact that the antibiotic is not necessary medically?	74	7	35	59	7	32	61	13	31	40	13	28	0.209
	63.8%	6.0%	30.2%	60.2%	7.1%	32.7%	58.1%	12.4%	29.5%	49.4%	16.0%	34.6%	
Q4. Do you prescribe antibiotics based on the guidelines recommended in your country and your own hospital?	80	5	31	56	6	36	76	12	17	37	13	31	**0.001**
	69.0%	4.3%	26.7%	57.1%	6.1%	36.7%	72.4%	11.4%	16.2%	45.7%	16.0%	38.3%	
Q5. Before receiving the culture results, do you prescribe empirical antibiotic therapy based on the common types of bacteria and the antibiotic resistance pattern of your hospital?	86	9	21	68	7	23	82	7	16	49	11	21	0.191
	74.1%	7.8%	18.1%	69.4%	7.1%	23.5%	78.1%	6.7%	15.2%	60.5%	13.6%	25.9%	
Q6. Can you distinguish colonization from infection based on antibiogram results?	72	15	29	61	14	23	78	10	17	49	8	24	0.312
	62.1%	12.9%	25.0%	62.2%	14.3%	23.5%	74.3%	9.5%	16.2%	60.5%	9.9%	29.6%	
Q7. To change the antibiotic regime due to the antibiogram, do you notice the history of receiving previous antibiotics?	101	5	10	77	9	12	84	10	11	59	6	16	0.172
	87.1%	4.3%	8.6%	78.6%	9.2%	12.2%	80.0%	9.5%	10.5%	72.8%	7.4%	19.8%	
Q8. To use broad-spectrum antibiotics, do you consult infectious disease specialists?	63	13	40	74	5	19	76	9	20	68	4	9	**0.001**
	54.3%	11.2%	34.5%	75.5%	5.1%	19.4%	72.4%	8.6%	19.0%	84.0%	4.9%	11.1%	

A, Strongly agree or Agree; B, Neither agree nor disagree; C, Disagree or Strongly disagree.Bold values represent the statistically significant results.

## Discussion

The increase in AMR is a major threat to public health in the 21st century. Medical professionals are the key stakeholders in the prevention and control of AMR through strict and effective prescribing practices, promoting awareness of ASPs and antibiotic resistance mechanisms, as well as they are the cornerstone of good health systems.

To the best of our knowledge, this is the first such study conducted among Iranian medical residents. The present study was conducted among the medical residents of Isfahan University of Medical Sciences toward AMR and MDR organisms and ASPs, as they are the key prescribers of antibiotics during their studies in medical-educational centers of Iran. There are few published studies around the world about the KAP regarding these topics among medical residents (15, 16). However, some previous studies evaluated the KAP in general practitioners, medical students and specialists (6–8). To date, there is no other study that attempted to address this topic in the country except one (7). It should be noted that this study is different from that paper since it assesses other issues in addition to the prescription of antibiotics and includes only medical residents.

This report revealed low levels of knowledge in the study respondents. The respondents scored on average 53.70 ± 15.88% correct answers regarding AMR and MDR organisms and ASPs. Similarly, a study showed that 55% the Chinese medical doctors had low levels of knowledge about antibiotic prescriptions (12). On the other hand, proper knowledge was observed in a study conducted among South Indian medical students (17).

Although knowledge of the ASPs is necessary for the residents, the majority of our respondents (73.2%) declared that they did not know what antimicrobial stewardship meant and it was not taught during the 4 years of their study because the knowledge has not changed in years. The average age of our statistical population was 30.9-year-old physicians who worked in hospitals and clinics before starting residency and unfortunately, despite working in the clinical environment, they did not know the program. The gaps identified in knowledge among our residents regarding ASPs require heightened educational interventions and these activities could be targeted. Therefore, we recommended reinforcing the curriculum by introducing more lectures on ASPs. Some countries, such as France, the United States and Scotland, have developed a national recommendations program to improve antibiotic stewardship in their countries (4).

Amazingly, only 45.5% of the respondents answered correctly that quinolones provide exceptional coverage against atypical pathogens. Notably, 28.6% of group 3 were aware that fluoroquinolones can cover the atypical bacteria, which is very low compared to other groups. It was expected that the knowledge would increase based on the years of education and that the first group compared to other groups would be different due to their daily involvement with antibiotics. Low levels of knowledge about the levofloxacin spectrum were documented among the clinicians in Pakistan and only 20% correctly identified it to be effective against atypical bacteria (4). Our result revealed that there were no significant differences in the knowledge among the residents based on the professional status of participants and participants’ years of entering the university. This is probably due to the lack of AMR knowledge transfer from professors to residents. In fact, the knowledge transfer chain is incomplete because these same residents become professors after graduation.

MRSA is an important human pathogen that causes a wide variety of community-and health care-associated infections. Accuracy and promptness in the detection of meticillin resistance using disk diffusion testing with oxacillin or cefoxitin are of fundamental importance in ensuring correct antibiotic treatment in patients and control of MRSA in the hospital (18). The results of our study showed that only 51.3% of our residents knew that cefoxitin disc is used to identify bacteria.

In a review study (19), it has been shown that coagulase-negative *staphylococci* are the most commonly isolated bacteria in blood cultures, and is mostly regarded as contamination. Therefore, differentiating infection from contamination in blood culture leads to the correct decision to prescribe antibiotics for the patient. In the present study, 29.2% of the residents were not aware that the *S. epidermidis* isolated from the blood culture may be contamination, which will lead to the incorrect prescription of antibiotics.

In our study, the majority of respondents 80 and 85.8% stated that antibiotics were used inappropriately in Iran and their workplace hospitals, respectively. This is consistent with previous studies (20, 21). In contrast, in Ghana, the majority of the respondent physicians answered that antibiotics were used appropriately in their units or departments (13).

The inappropriate use and overprescribing of antibiotics are a serious and long-standing challenge in Iran’s health system. According to the annual report of the health department of Iran’s Health Ministry, our country has reported high resistance rates in the bacteria of international concern such as MRSA (49%), vancomycin-resistant *enterococci* (VRE; 59%), *Escherichia coli* [resistance to third-generation cephalosporins (41%), or fluoroquinolones (54%)], *Klebsiella pneumoniae* [resistance to third-generation cephalosporins (83%), or carbapenems (69%)] and *Acinetobacter baumannii* [resistance to carbapenems (93%)]. Despite the restrictions to the initial prescription of some antibiotics such as carbapenems, colistin, linezolid and vancomycin which is a common measure in Iranian hospitals, we are witnessing an increase in antibiotic resistance day by day. It seems that Iranian residents have significant gaps in knowledge regarding AMR, ASPs and MDR pathogens especially extended-spectrum *β*-lactamase (ESBL)- and carbapenemase-producing bacteria. Our participant believed they were taught acceptably during their study about the interpretation of the cultures, the change of intravenous antibiotics to the oral types, and the mechanism of bacterial resistance. This is although, from their point of view, their information about how to start antibiotics was less. According to the reported knowledge, it does not seem that their attitude about their education was correct.

Thus, as with many other countries (9, 22, 23), it is crucial to develop appropriate curricula to teach the goals of ASPs, antibiotic resistance mechanisms, correct and prudent and timely antibiotic use to university medical residents. The percentage of residents giving the right answer for the management of ESBL bacteremia was 27.8%. Also, the low percentage (30.8%) of awareness about carbapenems and their resistance may be due to a lack of adequate education programs regarding the mechanisms of antibiotic resistance. A greater proportion (78%) of our respondents stated that they would prescribe azithromycin to a patient with symptoms such as sore throat, headache, and runny nose. This finding is consistent with previous study conducted in Zambia (21) that reported antibiotics can be used to treat cough, sore throat and colds. Our study showed that our residents were significantly associated with antibiotic use for cold. Although ceftriaxone is not effective in the treatment of cystitis, nor is azithromycin in the treatment of the common cold, a large percentage of our participants agree with prescribing these two. The indiscriminate prescription of these two common drugs can be a sign of the inappropriate prescription of antibiotics by physicians, which has led to the ineffectiveness of these two in the treatment of the disease.

Regarding the knowledge, the study found that the majority of the population (80.8%) stated that antibiotic resistance can impact their health and that of their families. A similar finding was found in a study conducted among the public in Cyprus, where 76.4% of respondents were concerned about the impact of resistance on the health of individuals and their families (24). Interestingly, the majority of our residents (72%) agreed that they did not get the necessary information about the right time to prescribe antibiotics during their university courses. Therefore, these results showed that there is a lack of sufficient training on antibiotic prescribing during education and pharmaceutical promotional activities in Iranian universities. It seems that prescribing antibiotics is a concern for residents and the need for infectious consultation to prescribe is felt, and they also try to use national and hospital guidelines.

Our study has some potential limitations. At first, it was conducted only at the Medical University of Isfahan and may not be a representative sample. Secondly, as with most surveys, there is the possibility that respondent residents obtained answers to some of the knowledge questions through Internet searches or literature reviews. Because in reality, it seems that their knowledge about some questions is less than the reported values.

## Conclusion

The present study highlighted the gaps in knowledge, attitude and practices of the residents regarding AMR, ASPs and MDR pathogens. The overall average knowledge score among residents was 53.70 ± 15.88. Group 1, which is the main antibiotic prescribing group, had an average score of 55.32 ± 16.10, which was not significantly different from the other three groups. As well, based on the year of entering the university, we see that the residents’ knowledge (Table 2) and attitude (Table 4) about the mentioned topics does not increase during 4 years, even in some cases, the knowledge has decreased. These findings indicate that education and intervention activities including reviewing the curricula and introducing the course of mechanisms of antibiotic resistance and ASPs in residents’ curricula are necessary to increase knowledge, promote responsible antibiotic use, and combat the growing threat of AMR.

## Data Availability

The raw data supporting the conclusions of this article will be made available by the authors, without undue reservation.
